# A Pegylated Flavin Adenine Dinucleotide PEG Complex to Boost Immunogenic and Therapeutic Effects in a Liver Cancer Model

**DOI:** 10.7150/ntno.59290

**Published:** 2021-04-22

**Authors:** Celia Arib, Hui Liu, Qiqian Liu, Anne-Marie Cieutat, Didier Paleni, Xiaowu Li, Jolanda Spadavecchia

**Affiliations:** 1 CNRS, UMR 7244, NBD-CSPBAT, Laboratoire de Chimie, Structures et Propriétés de Biomatériaux et d'Agents Thérapeutiques Université Paris 13, Sorbonne Paris Nord, Bobigny, France.; 2 Department of Hepatobiliary Surgery, Guangdong Provincial Key Laboratory of Regional Immunity and Diseases & Carson International Cancer Center, Shenzhen University General Hospital & Shenzhen University Clinical Medical Academy Center, Shenzhen University, Shenzhen, China.; 3 BioEVEN start-up, 75 rue de Lourmel 75015 Paris, France.

## Abstract

Flavin adenine dinucleotide (FAD) is engaged in several metabolic diseases. Its main role is being a cofactor essential for the activity of many flavoproteins, which play a crucial role in electron transport pathways in living systems. The aim of this study was to apply a pegylated flavins formulation named FAD-PEG diacide complex as theranostic pathway in cancer therapy. For this purpose, a mouse liver cancer model induced by Hepa1-6 cells was used to evaluate the therapeutic efficacy of FAD (named NP1) and FAD-PEG diacide complex (named NP2). The cytokines were applied to screen the serum inflammatory factors, to establish the blood cell content of different groups of nude mice. The highlights follows that FAD formulations (NP1; NP2) significantly suppressed the tumor growth and reduced the tumor index without effects on the body weight of mice. Furthermore, NP2 significantly reduced the serum levels of cytokines IL-6, TNF-α and IL-12 (P70). The reported results provide the proof-of-concept for the synthesis of a smart adjuvant for liver cancer therapy and support their further development in the field of nanomedicine.

## Introduction

Recent research studies demonstrate that human liver cancer is the fifth most common type of cancer. The percentage of liver cancer patients is highest in the uppermost in Asia and Africa, and conversely the lowest prevalence in Europe 1. The common type of liver cancer is hepatocellular carcinoma (HCC) 2. Some 75-90% of liver cancers are recognized as hepatocellular carcinoma (HCC) or malignant hepatomas, and it is the most frequent liver cancer 1. There are several limitations to conventional overtures for liver cancer therapy 2. Nanocarriers are capable of high drug loading capacity and stability, excellent tolerability, drug degradation, reduced multidrug resistance, controlled release and sustained delivery of anticancer drugs. Thus, therapeutics and theranostics drug delivery nanocarrier systems have significant benefits over common treatment methods 3,4. Nevertheless, there are many challenges needed to be resolved in drug delivery systems, such as undue accumulation of the carriers in the liver, poor therapeutic efficacy on the cancer cells and any barriers near the tumor areas or in the vasculature area that realize obstacles to penetration into liver cancer cells needed to be overcome 5. Besides, the controlled release of some drugs from its nanocarrier in liver cancer is another challenge in the development of nanomaterials that need further studies.

Flavin adenine dinucleotide (FAD) is committed in various metabolic reactions where the biological function is naturally connected to changes in conformation 6. Its primary role is being a cofactor necessary for the activity of several flavoproteins, which is important in electron transport pathways in many living systems 7,8. The last enzyme in the metabolic pathway producing FAD is FAD synthase, a protein confined in both cytosol and in mitochondria 9. Different regulatory processes for cell life and death, which are ROS production, antioxidant defense, protein folding, and chromatin remodeling, also depend on more than 100 different flavoproteins 10. Modifications in coenzyme levels have been remarked in many cancers 11. Coenzymes take part in regulating enzyme activity to perform disparate biochemical reactions 12. Mutations in metabolic enzymes obstruct typical biochemical reactions leading to many disorders 13,14,15.

Recently, J. Spadavecchia and D. Paleni have been conceived for the first time the role of FAD cofactor as hybrid theranostic complex in cancer therapy 16. The first aim of the present study, was to evaluate the evolution of novel adjuvant in chemotherapy by using FAD complexed to pegylated chaines to obtain hybrid complex (FAD PEG-Diacide) and combined with a common chemotherapeutic (5 Fluoro Uracile; 5-FU) in the therapy of liver cancer 17. For this purpose, a mouse liver cancer model induced by Hepa1-6 cells was used to evaluate the therapeutic efficacy of FAD (named NP1) and FAD-PEG diacide complex (named NP2). Animals were divided into 7 groups, which include: sham group (Sham), PEG group (Model), NP1 group (s.c.), NP2 group (s.c.), 5-FU group, NP1 and NP2 combined with 5-FU group. Hepa1-6 cells containing matrigel were inoculated under the second pair of breast pads on the left side of the mouse (the total number of injected cells was 1.2×10^7^), and administration was started 7 days after modeling, and the administration was continued for 3 weeks. All organs and tumor were dissected and the ratio (index) to weight was calculated. The cytokines were applied to detect the serum inflammatory factors, and the blood cell analyzer was used to determine the blood cell content of several groups of nude mice. The outcomes showed that FAD formulations (NP1; NP2) significantly suppressed the tumor growth and reduced the tumor index without effects on the body weight of mice. Moreover, NP2 meaningfully reduced the serum levels of cytokines IL-6, TNF-α and IL-12 (P70). We assumed that FAD PEG-Diacide boosts immune system and might influence the expression of 5-FU-associated enzymes and might increase sensitivity to 5-FU thanks to high metabolic stability under physiological conditions.

We believe that this study is crucial for planning optimal chemotherapeutic regimens and for understanding the mechanisms of adverse effects of 5-FU.

## Experimental section

### Materials and methods

Flavine Adenine Nucleotide (FAD) is provided by FISHER SCIENTIFIC at maximum purity grade. Dicarboxylic PolyEthylene Glycol (PEG)-600 (PEG) (PEG-Diacide), Sodium Chloride NaCl (0.9%), Phosphate-Buffered Saline (PBS), EDTA, 5 FluoroUracile (5-FU), Isoflurane, Paraformaldehyde were purchased by Sigma Aldrich at maximum purity grade. All solvents were used without any further purification. Experiments were carried out at room temperature if not specified otherwise.

HEMAVET 950FS animal blood analyzer special reagents (Drew Scientific, Inc., USA); Cytokine detection kit (Brand: Biolegend, USA). Cultrex®Basement membrane matrix high concentration, 10X (Manufacturer: Corning, USA). Coupling agent (300 mL) Manufacturer: Shandong Huikang Medical. 68.8%~69.8% electronic grade nitric acid; Electronic grade hydrochloric acid Manufacturer: South Korea's DUKSAM company; Tuning solution for ICP-MS: 7Li, 59CO, 115In, 238U Manufacturer: Thermo, 1.0 μg/L; Internal standard solution: 6Li, 45Sc, 72Ge, 89Y, 103Rh, 115In, 159Tb, 175Lu, 209Bi Manufacturer: National Nonferrous Metals and Electronic Materials Analysis and Testing Center, 1000 μg/mL; Gold single element standard solution Manufacturer: National Nonferrous Metals and Electronic Materials Analysis and Testing Center, 1000 μg/mL.

#### Instruments

Animal Weight Balance (Mettler - Toledo instruments (Shanghai) Co.,LTD*,* Serial number: PL3001-s), HEMAVET 950 Animal Blood Analyzer (Drew Scientific, Inc., USA- Model: HEMAVET 950FS), Automatic biochemical analyzer (Hitachi- Model: 7100), BD Accuri C6 Flow cytometer (BD company), High Resolution Small Animal Ultrasound Imaging System (Visual Sonics- Model: Vevo2100, probe MS400, frequency 30 MHz), Inductively Coupled Plasma Mass Spectrometer (ICAP-Q) (American Thermo Company).DB-3EFS type hot plate (Tianjin Gongxing Laboratory Instrument Co., Ltd), Milli-Q ultrapure water treatment system (American Millipore Company).

### Synthesis procedures of FAD-PEG Diacide Complex (NP2)

20 ml of FAD solution (c: 0.4 mM) was mixed with 500 µL PEG Diacide (1 mM) and stirring for 1 h at room temperature. After this time, the resulting yellow solution was sonicated for 2 h and purified by dialysis throught a membrane dialysis tubing Spectra/Por 3 (molecular weight cut-off 1500 Da, Serva Electrophoresis, Germany) with continuous stirring (80 rpm).

### Physical-chemical characterization

All characterizations were carried out in triplicate determinations as described previously 18.

### Dynamic light scattering (DLS) and Zeta potential measurements

The size and zeta potential measurements were performed by using a Zetasizer Nano ZS (Malvern Instruments, Malvern, UK) equipped with a He-Ne laser (633 nm, fixed scattering angle of 173°) at room temperature.

### FAD loading efficiency

The amount of the FAD incorporated into pegylated chaines was measured by UV-Vis absorption spectroscopy (**see S1 in Supporting Information**).

### Analysis LC-MS/MS

All samples are analysed with an UHPLC coupled with a Shimadzu triple quadripole LC-MS 8030 by ChemoBioFrance CNRS Platform.

### Stability in FAD (NP1) and FAD-PEG Diacide Complex (NP2)

The stability of **FAD (NP1)** and **FAD-PEG diacide complex (NP2)** were detected by UV VIS spectroscopy and UHPLC coupling to LC-MS**.** All Nanoparticles were dissolved in Dulbecco modified Eagle's Medium (DMEM) and stored for 72 h (**Figure S2 in Supporting Information**). The Stability of NP2 was also confirmed by UHPLC in plasma of mice during 120 min at 37 °C (1 µM, PBS, pH 7.4) (**Figure S3 in Supporting Information**).

### Metabolic stability of NP2 in hepatic microsomes

A 100 µM of NP2 solution is prepared by diluting the stock solution in water. This solution is diluted to 1/100 in a phosphate pad containing liver microsomes (0.5 mg/mL), 1 mM of NADPH and 3 mM of MgCl_2_. The final concentration in compound is 1M, the incubation volume is 400 µl the temperature is 37 °C. After two minutes of incubation, 70 µl are taken and mixed with 70 µl of maintained acetonitrile to stop the enzymatic reactions. 4 other samples, followed by the same treatment, are taken after 10, 20, 40 and 60 minutes of incubation. Negative control is carried out in parallel by replacing the NADPH co-factor with an equivalent volume to identify chemical instability or a non-addictive enzyme process NADPH co-factor. A sample is taken at t: 60 min only. A positive test is incubated under the same conditions: testosterone. All samples are frozen before analysis. After thawing, the samples are stirred with a vortex for 5 minutes, then placed in a ultrasound bath for 1 min and finally centrifuged for 5 min at 15,000 g at 16 °C. No degradation is observed after one hour of incubation.

The FAD-PEG (NP2) is stable in the presence of mouse liver microsomes. It is therefore not substrate of the CYP450 (**Figure S4 A-B in Supporting Information**).

### Hepa1-6 cells culture

Hepa1-6 cells purchased from the American Type Culture Collection (ATCC, Manassas, VA, U.S.A.) were grown in DMEM supplemented with 10% FBS, 2 mM glutamine, 100 U/mL penicillin, and 100 μg/mL streptomycin at 37 °C under a humidified atmosphere of 5% CO_2_.

### Antioxidant activity of NP1 and NP2

#### DPPH free radical scavenging assay

The percentage of antioxidant activity was assessed by DPPH free radical assay. An aliquot of 50 µL of the different concentrations of FAD (NP1) and FAD-PEG Diacide (NP2) (600, 280, 140, 72.5, 41.25 and 17.62 µg/mL) in water was added to 200µL of 0.254 mM DPPH in methanol solution and kept in the dark for 30 min at room temperature. The absorbance was measured at 450 nm against a blank of 30 µL DPPH in 200 µL methanol. Each measure was performed in triplicate and the DPPH free radical scavenging activity was calculated using the following equation:

DPPH scavenging effect (%) = [(A0 - At)/A0] × 100

A0 is the absorbance of the control and At is the absorbance of the sample.

### Mice tests

*In vivo* tests were performed using male nude mice (strain: BALB/cA-Grade: SPF; age 5 weeks; Mice production license number SCXK (Yue) 2018-0002, Guangdong Medical Laboratory Animal Center; Mice certificate number No. 44007200064015; No. 44007200070200; Mice Use License number SYXK (Yue) 2018-0001, Laboratory animal center, Guangzhou University of Chinese Medicine). The mices were fed in a specific room of SPF grade of Guangzhou University of Chinese Medicine. The nude mice had free access to food and water throughout the experiment and were housed under 12:12 h light/dark conditions in a temperature controlled environment (23 ± 3 °C) and the humidity being controlled at 40-70%. Experimental procedures were conducted in accordance with the NIH and were approved by the Experimental Animal Ethics Committee of Guangzhou University of Chinese Medicine.

After entering the SPF-level experimental center, the nude mice were quarantined in strict accordance with the relevant technical requirements of the SPF-level experimental animal center of Guangzhou University of Chinese Medicine. The quarantine time was 3 days. The general appearance and exercise conditions including state of consciousness, gait, response to stimulation, walking balance and limb coordination in rat were observed and recorded. Mices were randomly divided into seven groups: Sham group (n=10), PEG group (Model, n=7), NP1 group (FAD molecule) (s.c., n=8), NP2 group (FAD-PEG Diacide) (s.c., n=9), NP1+5-FU group (n=0), NP2+5-FU group (n=6), and the positive control 5-FU group (n=10).

Studies involving experiments with mice were in accordance with institution guidelines.

### Grouping and administration

Mice were divided into 7 groups (**Table 1**), which include: sham group (Sham), PEG group (Model), NP1 group (s.c.), NP2 group (s.c.), 5-FU group, NP1 and NP2 combined with 5-FU group. Hepa1-6 cells containing matrigel were inoculated under the second pair of breast pads on the left side of the mouse (the total number of injected cells was 1.2×10^7^), and administration was started 7 days after modeling, and the administration was continued for 3 weeks. After the formation of tumor model of nude mice (tumor volume reached 150-200 mm^3^, about 7 days), 100 µL of each group was injected intraperitoneally with 5-FU and continue to inject 100 µL NP1 and NP2 after 24 h, and given every 3 days for 3 weeks. The survival time of the tumor-bearing mice, body weight and tumors volume were measured and recorded.

### Detection indicators

#### General condition of body weight and organ index

The general situation of the mice including the activity, mental state, skin color, diet, water intake and urine output. Body weight (Bw) was weighed every 3 days. At the end of the experiment, the heart, liver, spleen, lung, kidney and tumor were separated and accurately weighed after the mouse were sacrificed, and the liver, spleen, kidney and tumor index were calculated (tissue weight (mg)/body weight (g)=Tissue weight/Bw).

#### Tumor volume detection

During the experiment, the length and width of tumor were measured using a vernier caliper at intervals of 3 days.

#### Whole blood cell count detection

Whole blood was measured with a blood cell counter on the 8^th^, 22^nd^, and 29^th^. Blood is collected in a tube containing EDTA, placed for 15-20 mins, and tested within 15 mins to 4 h. The optimal test time is within 30 mins to 2 h, which can be longer for healthy animals.

Samples of whole blood were sampled on EDTA tubes and analysed by means of an automated HEMAVET950FS animal blood analyzer to determine the concentrations of white blood cell (WBC), red blood cell (RBC), haemoglobin (HGB), hematocrit (HCT), mean corpuscular volume (MCV), mean corpuscular haemoglobin (MCH), mean corpuscular haemoglobin concentration (MCHC), platelets (PLT), Lymphocytes (LY), monocyte (MO), neutrophil granulocyte (NE), platelet volume distribution width (PDW), mean platelet volume(MPV), and platelet larger cell ratio (P-LCR).

### Multiplex bead-based assay for pro-inflammatory cytokine

Flow cytometry was used to determine serum cytokine levels on the 8^th^, 22^th^, and 29^th^. The blood was left to stand at 4 °C for 2-3 h or at room temperature for 1 h, and then centrifuged at 3500 rpm for 10 min at 4 °C. The supernatant was collected and stored in aliquots. During the measurement, the serum was diluted 2 times with assay buffer for subsequent flow cytometry detection.

### Sample preparation

**A.** Add 250 μL of assay buffer to the standard to form a liquor of 10000 pg/mL, and leave it at room temperature for 10 mins;

**B.** Take 8 tubes, numbered C1-C7, add 75 µL of assay buffer to each tube, and then add 25 µL of standard stock solution to C1 tube. After vortexing, transfer 25 µL to C2 tube. By analogy to C6, 75 µL is retained per tube.

### Flow cytometry

**A.** Pre-mixed the beads for 30s, mix the beads, and then dilute the beads 5 times with assay buffer for use;

**B.** Dilute 20 X washing buffer to 1 X washing buffer with deionized water for later use;

**C.** Take 5 mL of legend plex assay buffer into lyophilized matrix B and resuspend for 15 min at room temperature until use;

**D.** For standard samples, add 25 µL of matrix B solution, 25 µL of diluted standard solution, and 25 µL of pre-mixed beads in order to the tube; for the sample to be tested, add 25 µL to the tube in order. Assay buffer: 25 µL of diluted serum and 25 µL of premixed beads;

**E.** Incubate at room temperature for 2 h in the dark; then add 100 µL of washing buffer to each tube, centrifuge at 1000 rpm for 5 min at 4 °C, carefully remove the supernatant, and repeat this step;

**F.** Add 25 µL biotinylated detection antibody (5 times dilution in assay buffer) to each tube. Incubate for 1 h in the dark under temperature;

**G.** Without washing, directly add 25 µL of SA-PE reagent (5 times dilution of assay buffer), incubate for 0.5 h in the dark at room temperature, add 200 µL of 1X washing buffer and wash twice, discard the supernatant, and add 100 µL of 1X washing. The buffer is resuspended and detected on the machine.

### Statistical analysis

Statistical analysis of the experimental data was performed by GraphPad Prism 8.0 software. The data were expressed by Mean ± SEM. One-way ANOVA and Tukey test methods were used for comparison between groups. Results were considered statistically significant for *p*<0.05.

All experimental *in vivo* studies and pharmacodynamic experiments were validated by Chemo Bio France platform CNRS.

## Results and Discussion

This study is issued from a recently patent 16, in which we proved, for the first time, the power of FAD PEG-Diacide (NP2) as adjuvant in chemotherapy in term of stability, antioxidant activity and anti-tumor efficacy. For this purpose we conceived a lot of experiments in which NP2 was tested as pegylated complex and compared with control (FAD; i.e. NP1) and anti-cancer drug (5-Fluoro-Uracile [5-FU]).

### Plasmatic and metabolic stability of FAD PEG-Diacide (NP2)

The microsomal stability assay is mainly used to inquire Phase I metabolism using NADPH as the enzyme co-factor 19. Metabolic stability is determined as the percentage of parent compound lost over time in the presence of a metabolically active test system 20. For metabolic stability assays, the typical test systems are liver microsomes, liver S9, or hepatocytes (plated or suspended), depending on the goal of the assay. To realize the metabolic stability of a new chemical entity, quantification of the drug candidate from incubate supernatants is required and usually accomplished by high-performance liquid chromatography (HPLC) with mass spectrometry. At the first time, analysis was performed in cell culture medium (Dulbecco's Modified Eagle's Medium-DMEM) over a period of 144 hours. FAD PEG-Diacide (NP2) exhibited no change in the UV VIS spectra after 72 h (**Figure S2 in Supporting Information**) indicating that NP2 are highly stable and that their size remains unchanged during the time. Zeta potential measurements confirmed the spectroscopic results, showing that NP2 solution is stable at physiological pH (z-potential = -31 ± 1 mV d: 152 nm with a PdI equal to 0.259±0.002). We assume that NP2 stability is due to the presence of the PEG polymer chains. The stability in DMEM was also confirmed by measurements in plasma of mice for 120 min at 37 °C (PBS, pH 7.4) (**Figure S2 in Supporting Information**) and in microsomes of liver mouse during 1h at 37 °C (PBS, pH 7.4) in presence of NADPH cofactor (**Figure S4 A-B in Supporting Information**).

On the basis of these findings we assumed that the complex FAD-PEG-Diacide (NP2) was not a substrate for CYP450 21,22 and consequently a good candidate as adjuvant and anticancer drug.

### Antioxidant activity

Oxidative stress plays a key role in the pathogenesis of several human disease states including cancer, diabetes and angina pectoris 23. Oxidative stress is responsible of degenerative diseases along with aging. It was proved that FAD (NP1) is necessary for glutathione reductase (GR) enzyme to convert oxidized glutathione (GSSG) to the reduced glutathione (GSH) as an endogenous antioxidant in different cells 24.

The antioxidant activity of FAD (NP1) and FAD-PEG Diacide (NP2) was assessed by DPPH method 25.

#### Adjuvant chemoterapy and synergic therapeutic effect: Hypothesis

5-Fluorouracil (5-FU) has widely been applied as an anticancer drug over the past five decades 26, 27. 5-FU is cell-cycle inhibitor, which specifically inhibits at the S phase; it is an analogue of uracil, by including into DNA or RNA, which provoke cytotoxicity of the cells and cell death 28, 29. Upon the entry of 5-FU into the cell, it is transformed into fluorodeoxyuridine monophosphate (FdUMP), followed by preventing the DNA replication by interacting with the thymidylate synthase. Some reports suggest that 5-FU induces cell death by the caspase-6 pathway activation, phosphorylating Bcl2 protein, and improved the accumulation of mitochondrial ROS 30, 31. The intracellular derivative of 5-FU and 5-fluorouridine-5′-triphosphate (FUTP) can also incorporate into RNA synthesis 32. Previously, Barile et al. proved the presence of FAD protein in the nuclei of primary cells and cell lines by classical immuno-based techniques and its functionality in intact nuclei from rat liver by biochemical assays 33. FAD localizes not only in the cytosol but also in mitochondria and nuclei, as already suspected when observing the corresponding immuno fluorescence results described above 34. Whichever the isoform details, to our acquaintance this is the first evidence that FAD synthesis come off in the nucleus, and we assumed that, as hypothesized by others 34, this event could be related to the biogenesis and flavinylation of nuclear flavoproteome.

**Scheme 1:**
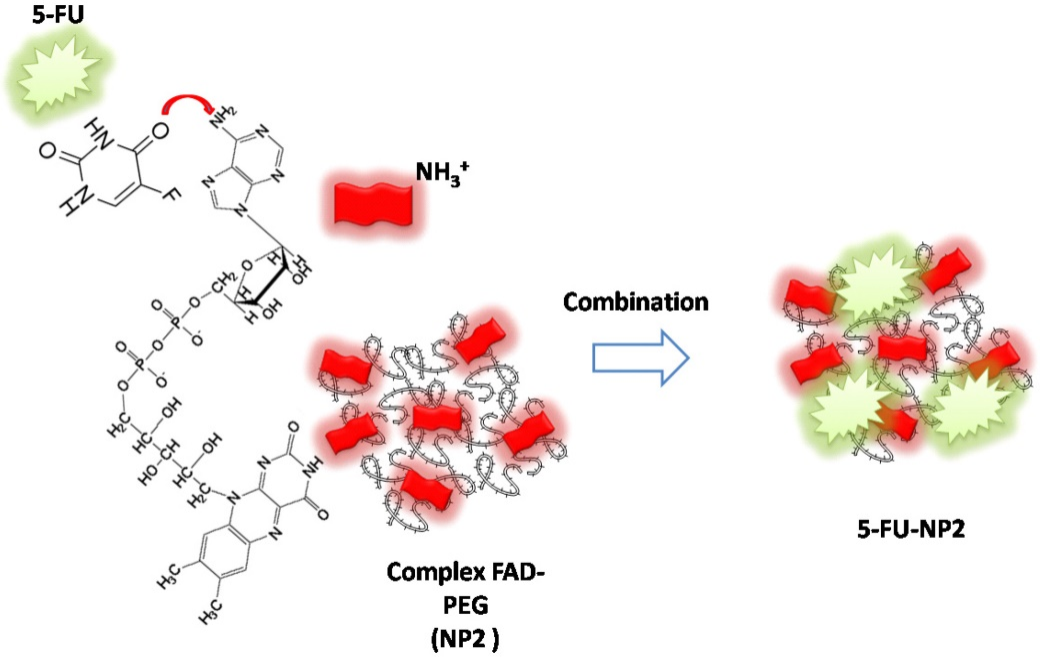
Schematic representation of chemical combination between 5-FU and FAD PEG Diacide (NP2) to form hybrid chemotherapeutic mixture (5-FU-NP2).

**Scheme 2:**
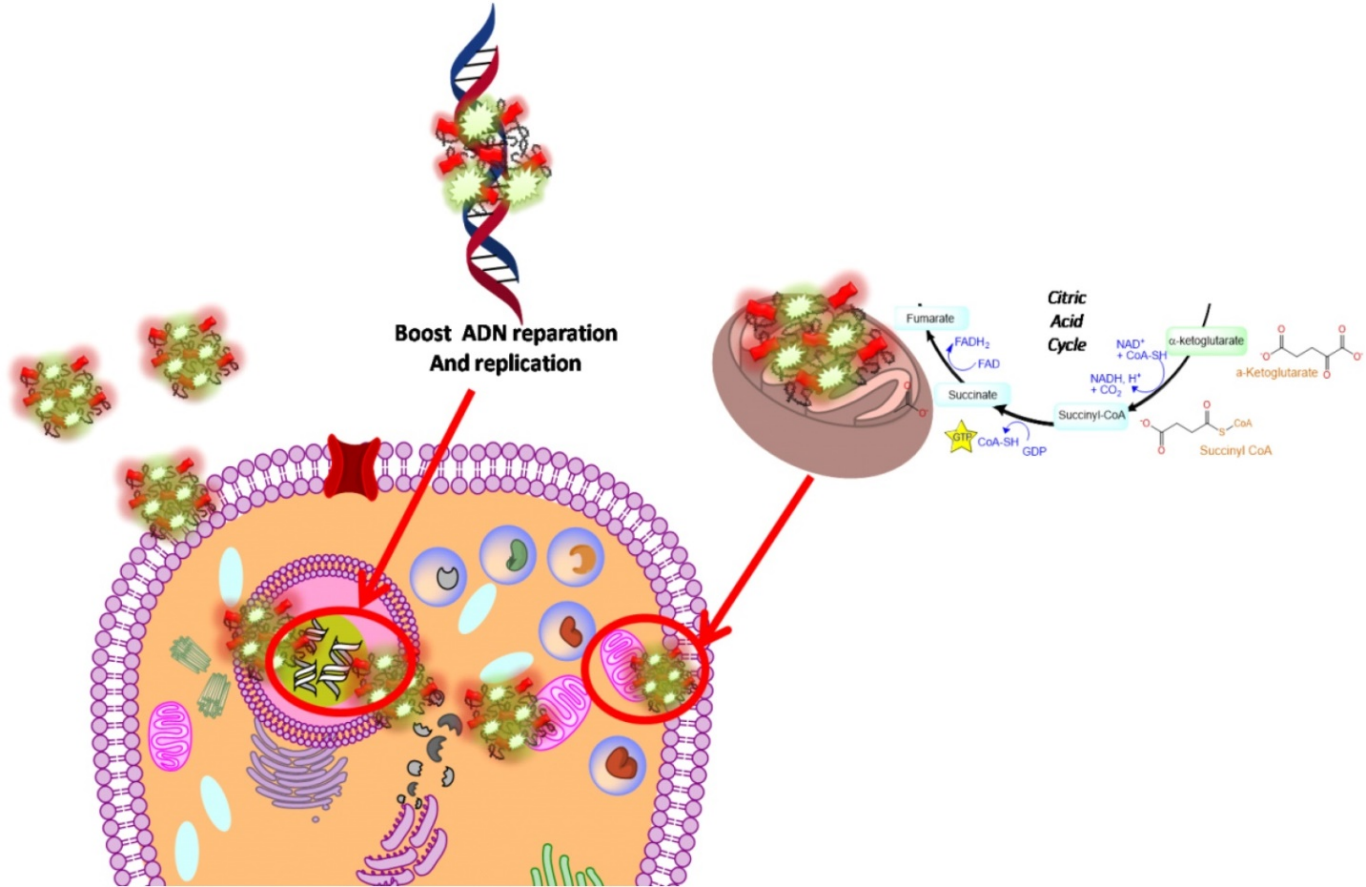
Schematic hypothesis of 5-FU-NP2 effects after cancer cell internalization.

In our paper, we combined FAD PEG-Diacide (NP2) with 5-FU crosswise electrostatic charges between the chemical groups of the drug and NP2 under specific conditions (**Scheme 1**). Indeed it was established the 5-FU have an amino group positively charged in water that interacts with the carboxylic group of the polymer (PEG-diacide) of NP2 with a consequently steric and chemical modification of both compounds (FAD PEG-Diacide (NP2); 5-FU) in the performed hybrid mixture (5-FU-NP2). In this chemical configuration, we assume that 5-FU-NP2 under complex form, after internalization in cancer cell, synergic improve the therapeutic efficacy, thanks a double action on nuclei and mitochondria (**Scheme 2**). Infact FAD and 5-FU were released in the cytoplasm as pegylated complex through migration of 5-FU and FAD in the PEG chains, blocking the binding of aminoacyl-tRNA to the mRNA-ribosome complex with evident variation in driven force and electrolytic condition. Consequently, the synergic effect of FAD and 5-FU as complex, under different steric conformation, improve the inhibition of Glucoso 6 phosphate dehydrogenases 35 and dihydropyrimidine (DPD) 36 with a consequently simultaneous reparation, and inhibition of resistance and improvement of anticancer activity.

### *In vivo* antitumor efficacy

**Figure 1:**
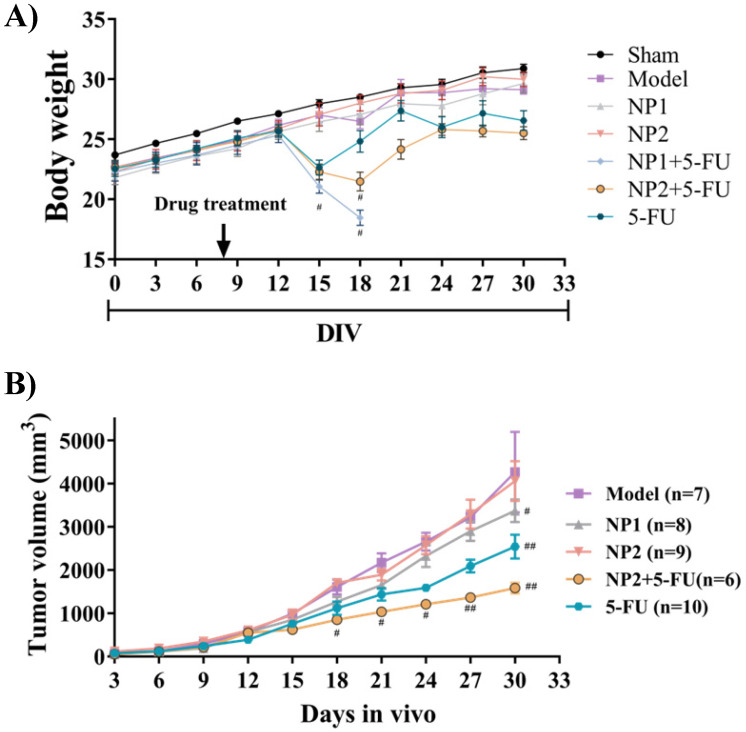
** A)** Effect of NP1 and NP2 on body weight change in liver mice with Hep1-6 cells injection. Body weight was shown as Mean ± SEM. *#P* < 0.05 vs model group. **B**) Effect of NP1 and NP2 on tumor volume in liver mice with HEP1-6 cells injection. Values were shown as Mean ± SEM. *#P*< 0.05 and *##P*< 0.01 vs Model group.

The therapeutic effect of FAD-PEG Diacide (NP2) compared to FAD (NP1) on liver cancer, was evaluated using nude mice in which human Hepa1-6 cells were hypodermic injected to generate a subcutaneous liver cancer. The mice were weighed every 3 days. As shown in **Figure 1A**, the weight of the mice in the model group displayeda slight decrease compared with the sham group. Compared with the model group, NP1 and NP2 did not affect the weight of the liver cancer model mice. However, the positive control drug 5-FU and NP2 combined with 5-FU group, showed a significant decrease in body weight at the end of the experiment. NP1 combined with 5-FU group began to lose weight on the 12^th^ day, and all mice died after the 18^th^ day.

### Tumor, liver, spleen, thymus and kidney index detection

**Figure 2:**
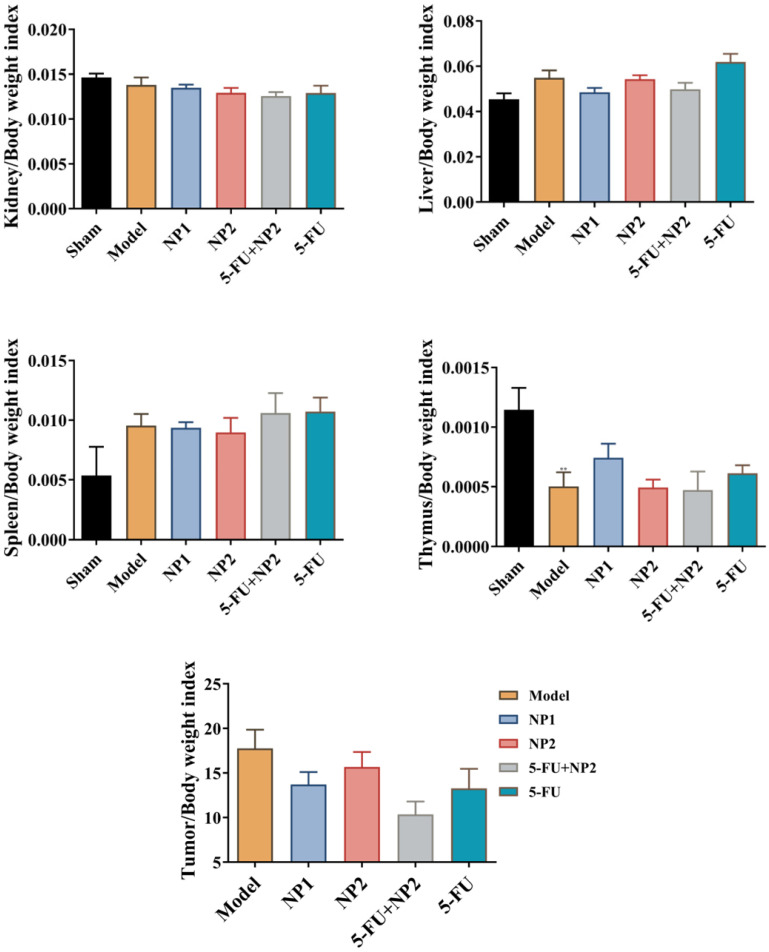
Effect of NP1 and NP2 on tissue index in liver mice with Hepa1-6 cells injection. Values were shown as Mean ± SEM. **p < 0.01, model vs sham.

The tumor length and width of the mice were measured every 3 days (**Figure 1B**). The tumor volume in the model group gradually increased. The NP1 group, NP2 combined with 5-FU group, and the 5-FU group as control could delay the tumor volume increase in mice. NP1 group, NP2 combined with 5-FU group and 5-FU group had significant statistical significance (p<0.05). More importantly, the treatment effect of NP2 combined with 5-FU group was better than the positive control drug 5-FU. At the end of the experiment, the liver, spleen, kidney, tumor and thymus of each group of mice were dissected and separated, and the weight ratio of the mice to the corresponding mice was calculated (**Figure 2**). All images are shown in **Figure S5 in Supporting Information**. The liver and kidney indexes of mice in different groups were not statistically different. Compared with the sham group, the spleen index of the model group increased and the thymus index decreased; compared with the model group, the NP1 group could increase the thymus index and decrease the tumor index, but does not affect the spleen index. A very strong reduction of tumor was observed when NP2 is combined with 5-FU group with a consequential improvement of treatment. This behavior is probably due to specific chemical affinity of FAD conjugate to PEG Diacide to 5-FU, which induce a better steric arrangement and a consequent better therapeutic effect.

### Effects of NP1 and NP2 on blood cells

It was discussed previously 37 that the higher concentration of FAD in Glucose-6-Phosphate Dehydrogenase (G6PD) deficient erythrocytes is due to a rapport between the increased activity of Glutathione reductase (GR) in G6PD deficient cells, and the high level of FAD concentration 35. However, the binding of FAD by GR is inhibited by certain nueleotides, e.g. NADPH and ATP 38, and low concentration of these nucleotides in G6-PD deficient red cells 39,35 may provoke in increased binding of FAD by GR. If FAD in erythrocytes occurs mainly or exclusively bound to GR the increased binding itself may be the cause of the higher concentration. In our study, blood cell counts were used to measure the number of cells in whole blood after 1 w (DIV 8), 3 w (DIV 22), and 4 w (DIV 29) after administration. As shown in **Figure S6 in Supporting Information** after 4 weeks of administration, NP1 group increased the number of WBC, NE, MO and EO which is in line with the features of common chemotherapy drugs. Meanwhile, NP2 combined with 5-FU group significantly increased WBC, NE, LY, MO in different degrees, EO and BA numbers compared with 5-FU group. We presume that in NP2 formulation, the high protection and steric arrangement of FAD molecules in pegylated chains, allows a better chemical interaction with 5-FU that permit a boost enhancement of blood cells that provoke inhibition of G6PD.

### Effects of N1 and N2 on cytokines expression

Cytokines and chemokines are signaling proteins produced from several types of cells that regulate immune responses 40. There are two immune responses for cytokine production: 1) antigen presenting cells (APCs) take up antigens, process them, and subsequently show them to T-lymphocytes to produce cytokines, and; 2) APCs, such as monocytes, are activated to produce cytokines through pattern recognition receptors that identify a foreign pathogen 41. Cytokines predominantly produced by APCs include multiple interleukin (IL) and tumor necrosis factor (TNF) molecules 42. Different clinical studies have showed that administering a standard dose of chemotherapeutic drugs induces an increase in cytokine levels for a variety of cytokines (TNF-a, IL-6, IL-8, IL-10, and monocyte chemotactic protein-1 [MCP-1]) 43. Cytokines normally function to improve a host response aimed at cellular stress and minimizing cellular damage. The failure to solve an injury can produce excessive immune cell infiltration and lead to persistent cytokine production. The liver hosts many cell types that are sensible to the actions of cytokines 44. Hepatocytes bear a kind of cytokine receptors such as IL‐1, TNF‐α, and IL‐6 45. Cytokines have thus been involved in liver development and regeneration but may also contribute to the pathogenesis of liver‐related diseases such as cirrhosis, fibrosis, and cancer.

A significant problem in oncology clinics is the cytotoxic effect of cancer chemotherapy on gastric cancer 46. For example, mucositis is a relevant oncological problem caused by the cytotoxicity of chemotherapy 43, 47. The antimetabolite agent 5-fluorouracil (5-FU) has been used in the treatment of a range of cancers and provokes intestinal damage, referred to as intestinal mucositis 47. Soares et al. showed that in animal models, intestinal mucositis induced by 5-FU is related with neutrophil infiltration, increased pro-inflammatory cytokine levels and, importantly, delayed gastric emptying 48.

Williams et al. 49 reported that the inflammatory cytokines TNF-α and IL-β contribute to the severity of intestinal mucositis.

Here we reported the incidence of NP1 (FAD) and NP2 (FAD-PEG Diacide) onto cytokine level and the synergic effect due to the combination between NP2 and 5-FU, commonly used in chemotherapy.

**Figure 3:**
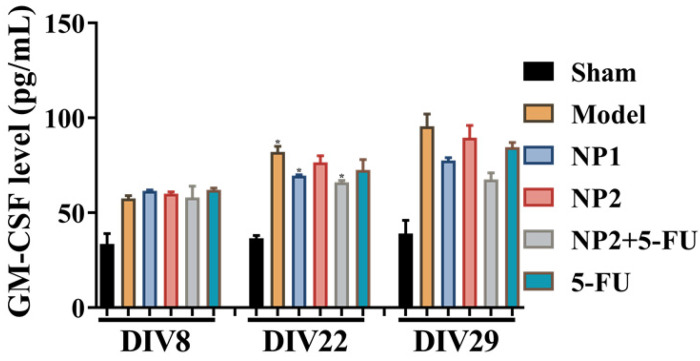
Effect of NP1 and NP2 on serum cytokine in liver mice with HEP1-6 cells injection. *p<0.05 vs sham group.

As shown in **Figure 3**, the content of IFNβ, MCP-1, IL-27 and IL-17A in the corresponding time points were not different between different groups. After 3 and 4 weeks of administration, compared with the sham group, the levels of IL-1α, IFNγ, TNFα, IL-1β, IL-6, IL-12P70 and GM-CSF in the model group increased, and decreased IL-10 level. Compared with the model group, the NP1 group reduced the contents of IL-1α, IFNγ, TNF α, IL-1β, IL-6 and GM-CSF, and increased the content of IL-10. Meanwhile, NP2 combined with 5-FU group reduced the levels of IL-23, IL-1α, IFNγ, TNFα, IL-1β, IL-6 and GM-CSF. The anti-inflammatory effect of NP2 combined with 5-FU group is better than that of 5-FU group and NP2 group.The synergic combination within NP2 (FAD-PEG Diacide Complex) and 5-FU significantly reduced the serum levels of IL-6, TNF-α and IL-12 P70 due to anti-inflammatory properties of FAD 50. Besides NP2 + 5-FU decreases dramatically the level of GMF-CSF (Granulocyte Macrophage Colony Stimulating Factor), a white blood growth factor 51. The decreases of GMF-CSF results in less infection compared with other groups, this is mainly due to formulation who protect the immune system and could be collapsed by the drug and tumors (**Figure 3**).

**Scheme 3:**
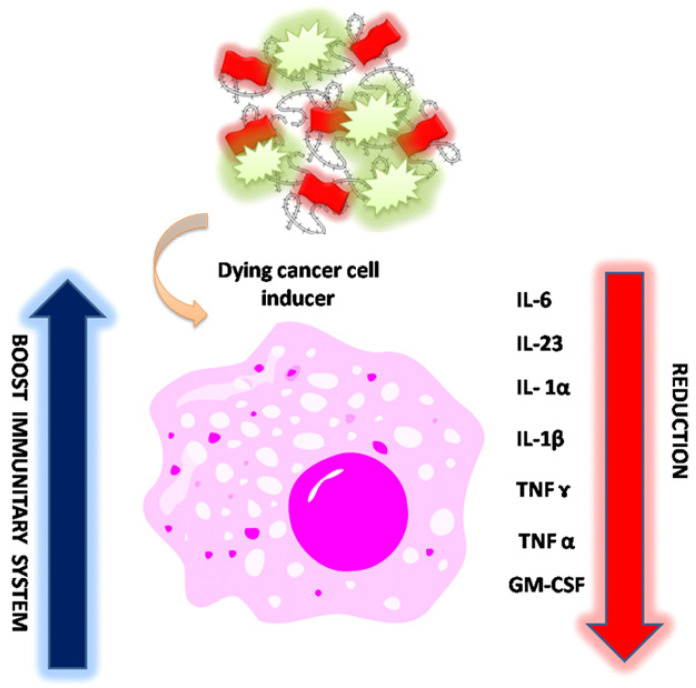
Schematic effect of NP2-5-FU onto cytokine level after cancer cell induction (all drawings are not in scale).

On the basis of these findings, we assume that NP1 and NP2 decrease the level of pro-inflammatory cytokines and the synergic combination between NP2 and 5-FU strongly improve immune system effects (**Scheme 3**), due to the activation of calreticulin and consequently decrease of serum level of IL-6, IL-12, TNF-α and CM-CSF 52.

## Conclusions

**Scheme 4:**
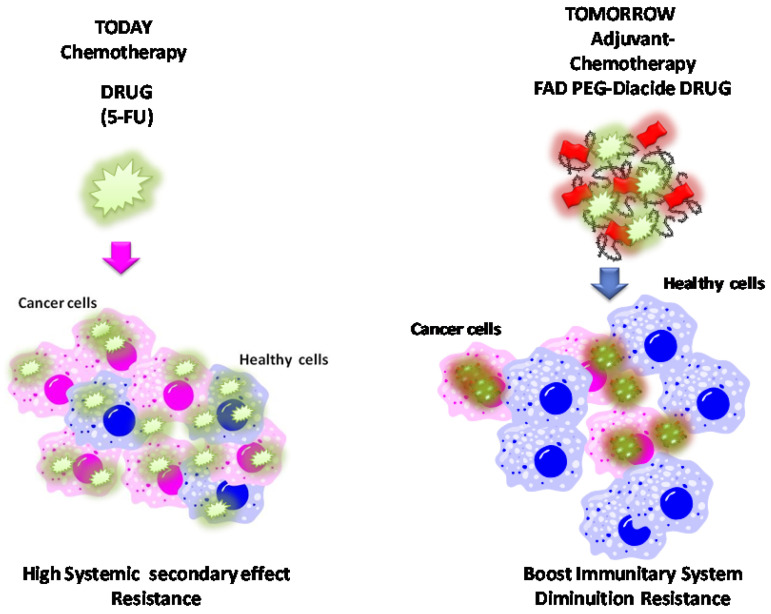
Schematic comparison among traditional chemotherapeutic drug on today (left panel) and future prediction of FAD-PEG Diacide onto cancer cells as adjuvant in chemotherapy tomorrow (right panel).

In this paper, we have explored the chemo-biological relevance of Flavins coenzymes as biocompatible formulation in cancer metabolism, adjuvant and anticancer drug. The advanced antitumor efficacy of FAD PEG-Diacide (NP2) compared to 5-FU displays not only in the repression of tumor growth but evenly in higher stimulation of immune system. The signal pathway of NP2 acting on blood cells/immune cells/tumor cells and the analysis of tumor tissue or immune cells will be applied in genomics and proteomics. These results predict the wave for the development of an innovative theranostic platform, allowing the detection of protein-associated tumors and the simultaneous cancer treatment with an adjuvant that reduce secondary effect of chemotherapy and boost immunitary system with a consequent good replication of healthy cells (**Scheme 4**). This paper provides the proof-of-concept for the synthesis of a smart adjuvant for liver cancer therapy and supports their further development in the field of Cancer Therapy. We currently study our system to develop a clinical Phase I thanks to good metabolic stability.

## Supplementary Material

Supplementary figures.

## Figures and Tables

**Figure 4:**
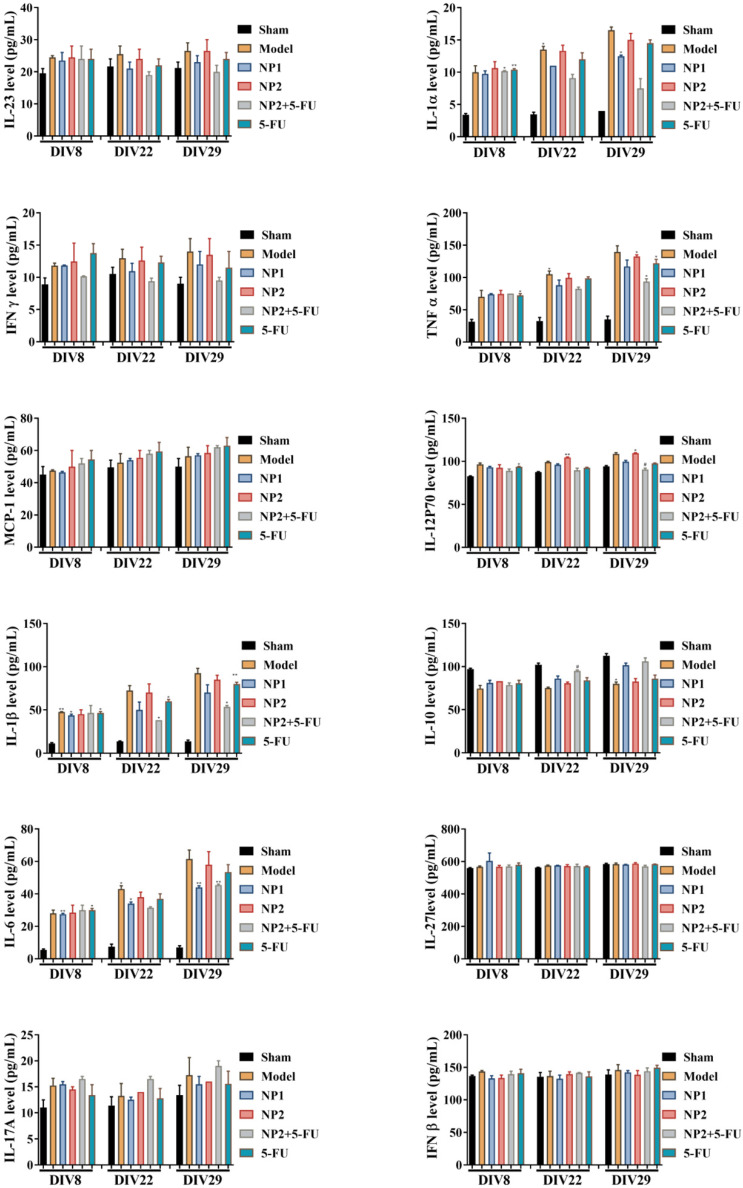
Effect of NP1 and NP2 on serum cytokine in liver mice with HEP1-6 cells injection. *p<0.05, **p < 0.01 vs sham group; *#p*< 0.05 vs Model group.

**Table 1:**
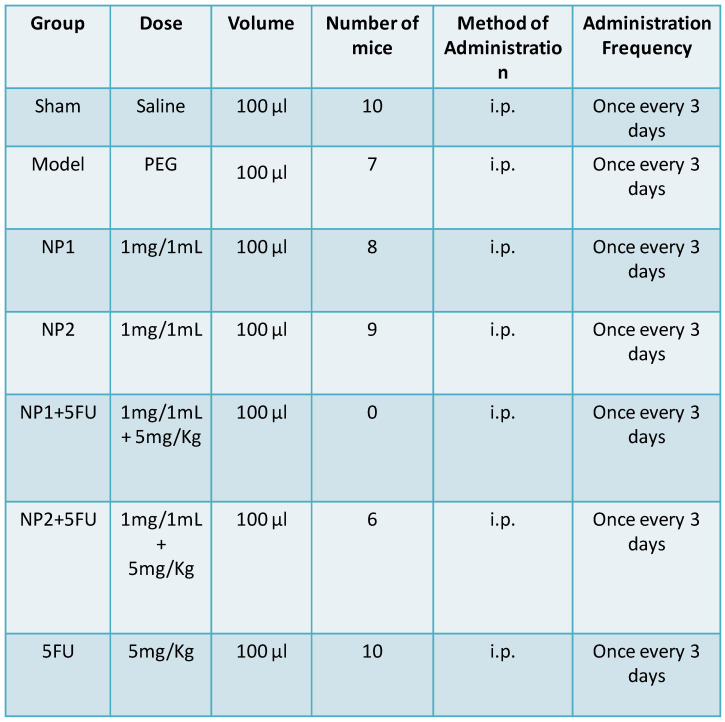
Experimental conditions of FAD formulations (NP1; NP2) administration
