# Supplementary material for: A Pegylated Flavin Adenine Dinucleotide PEG Complex to Boost Immunogenic and Therapeutic Effects in a Liver Cancer Model
Source: Nanotheranostics. 2021 Apr 22;5(4):405–16. doi: 10.7150/ntno.59290 (PMC8077970; doi:10.7150/ntno.59290)
Supplement: Supplementary file 1 — Supplementary figures. [file ntnov05p0405s1.pdf]

-SUPPORTING INFORMATION-

A Pegylated Flavin Adenine Dinucleotide PEG Complex to Boost Immunogenic and Therapeutic Effects in a Liver Cancer Model

Celia Arib[1‡], Hui Liu[2‡], Qiqian Liu[1], Anne-Marie Cieutat[1], Didier Paleni[3], Xiaowu Li[2*], Jolanda Spadavecchia[1-2*]

[1] CNRS, UMR 7244, NBD-CSPBAT, Laboratoire de Chimie, Structures et Propriétés de Biomatériaux et d'Agents Thérapeutiques Université Paris 13, Sorbonne Paris Nord, Bobigny, France

[2] Department of Hepatobiliary Surgery, Guangdong Provincial Key Laboratory of Regional Immunity and Diseases & Carson International Cancer Center，Shenzhen University General Hospital & Shenzhen University Clinical Medical Academy Center, Shenzhen University, Shenzhen, China

[3] BioEVEN start-up, 75 rue de Lourmel 75015 Paris

Corresponding Author*: lixw1966@163.com; jolanda.spadavecchia@univ-paris13.fr

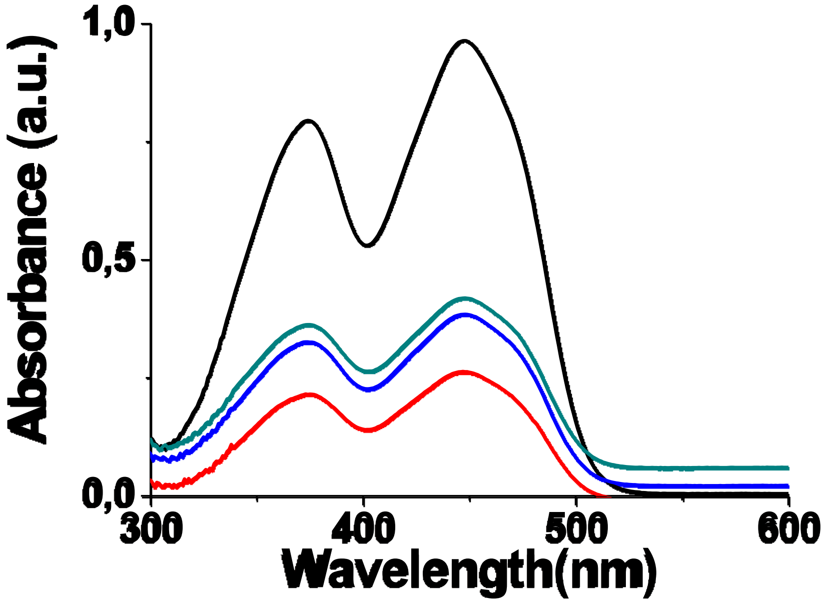

Figure S1: UV-Vis absorption spectra of (A) FAD at increasing known concentrations.

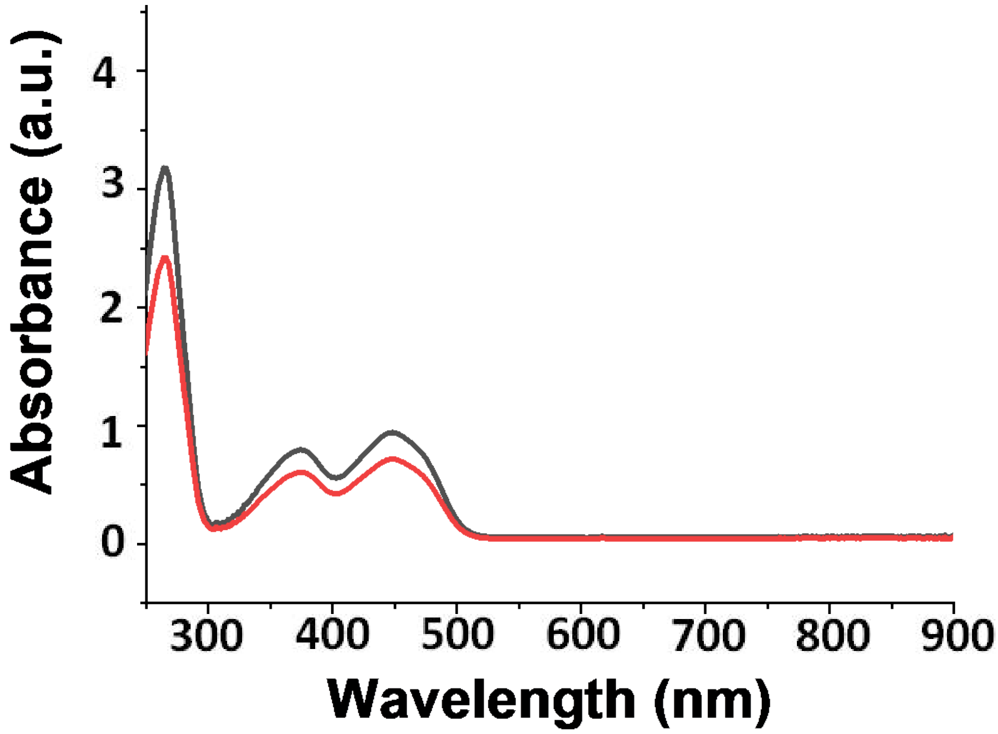

Figure S2. Stability of FAD PEG-Diacide (NP2) before (black line) and after (red line) incubation in DMEM(72h).

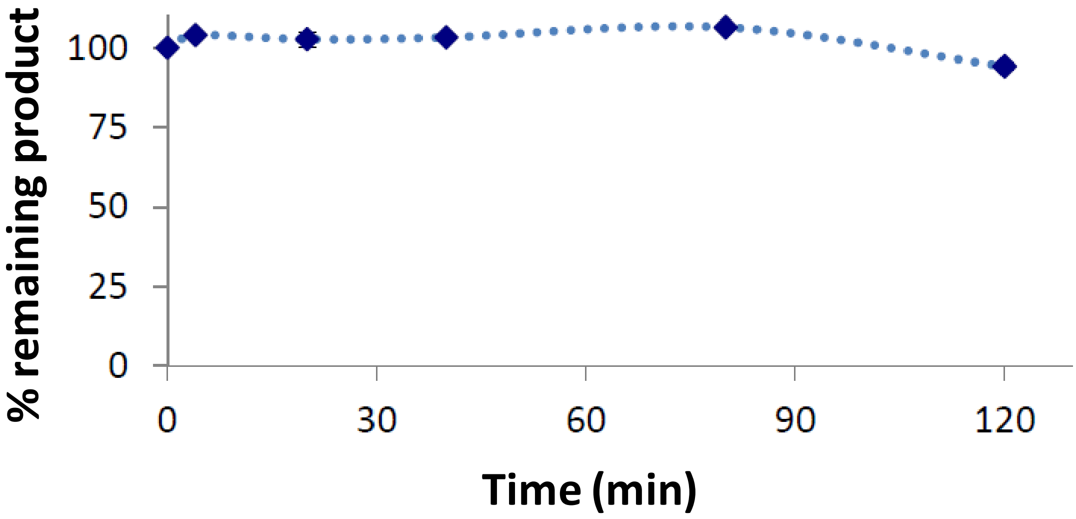

Figure S3. Stability of NP2 in plasma of mice during 120 min at 37°C (PBS, pH 7.

A)

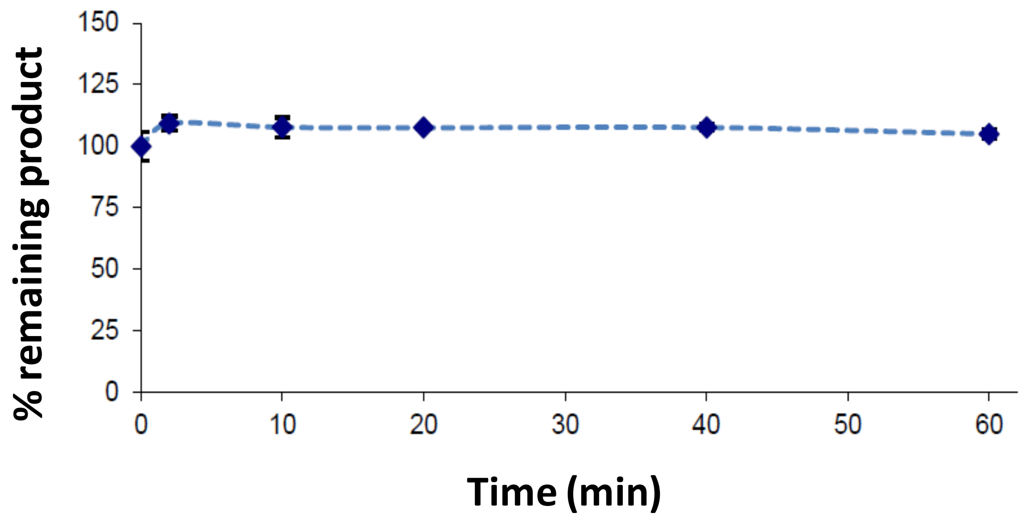

B)

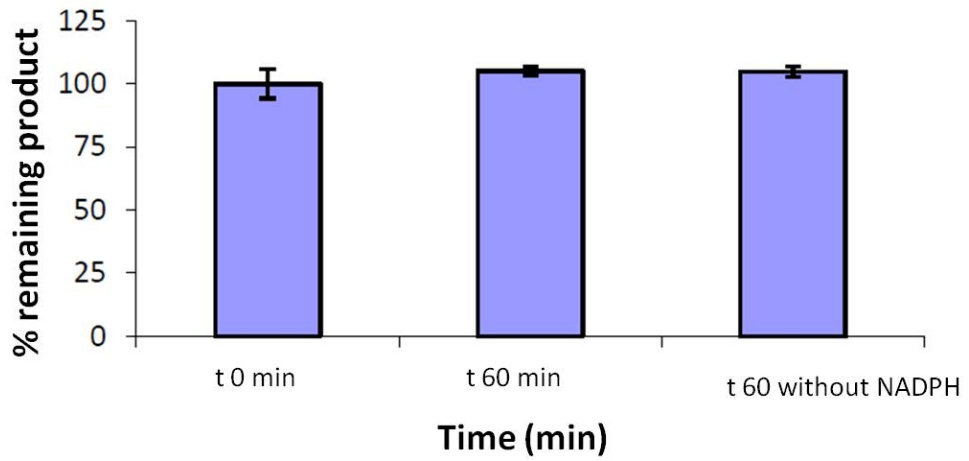

Figure S4.A) Stability of NP2 in microsomes of liver mouse during 1h at 37°C (PBS, pH 7.4) in presence of NADPH cofactor (B).

A)

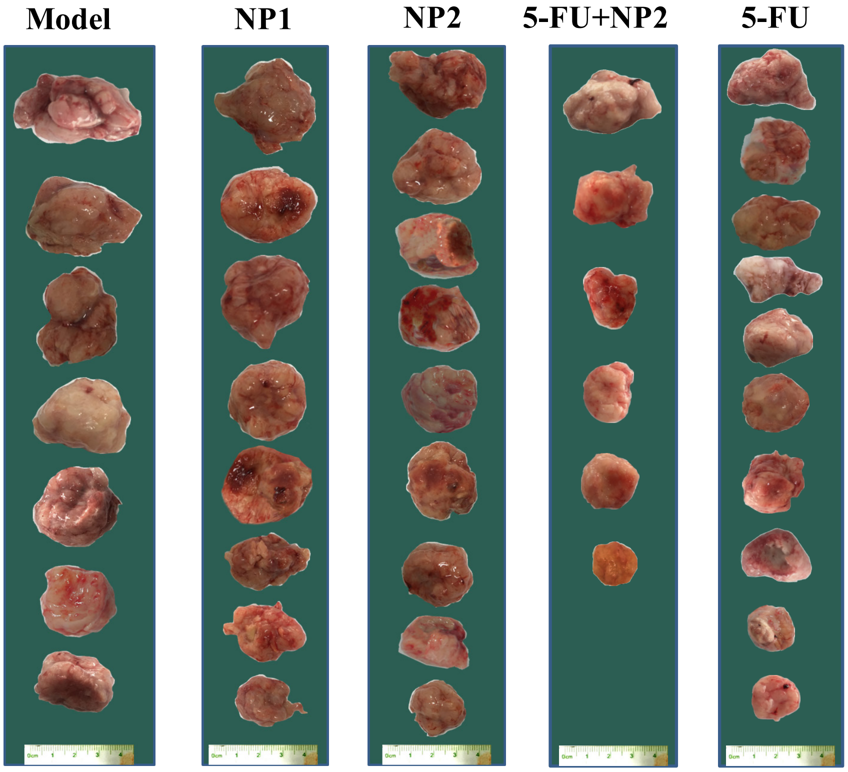

B)

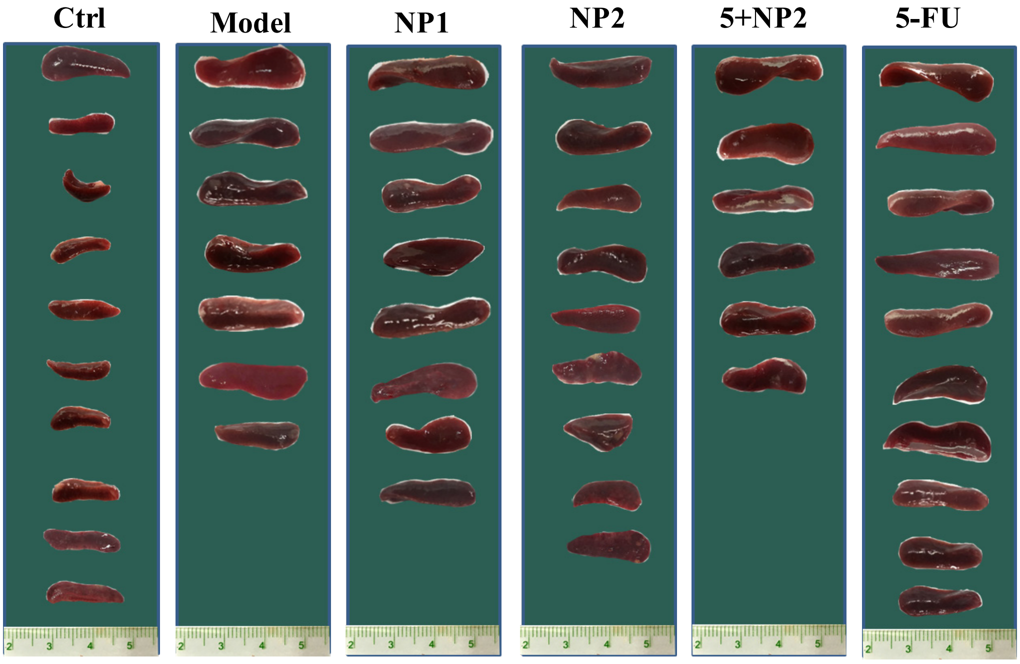

C)

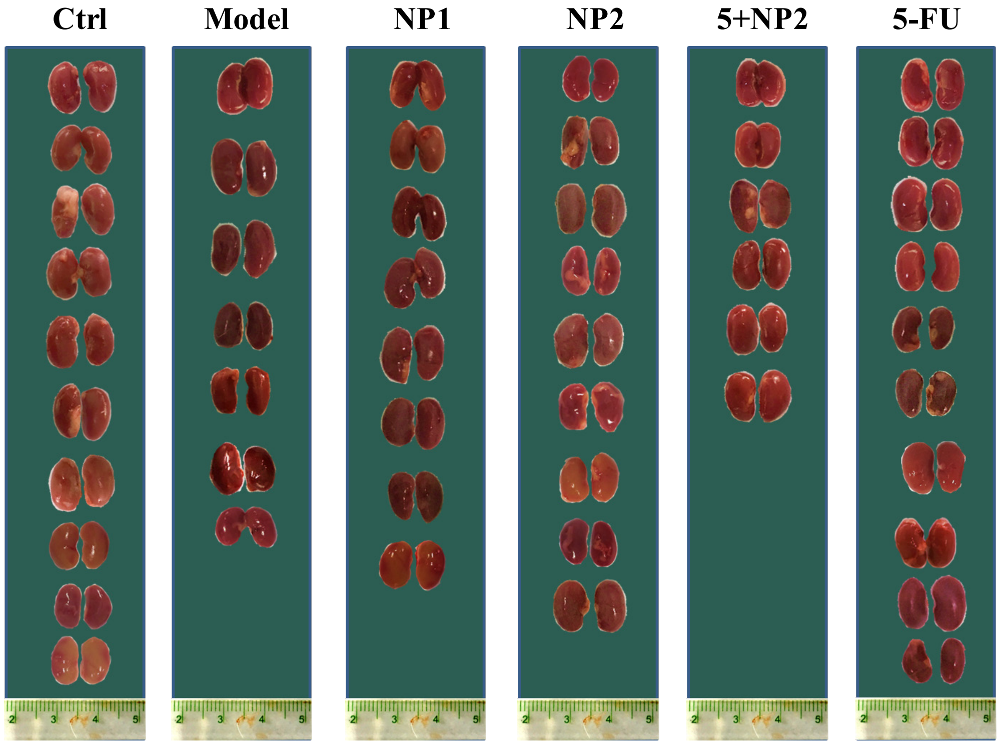

D)

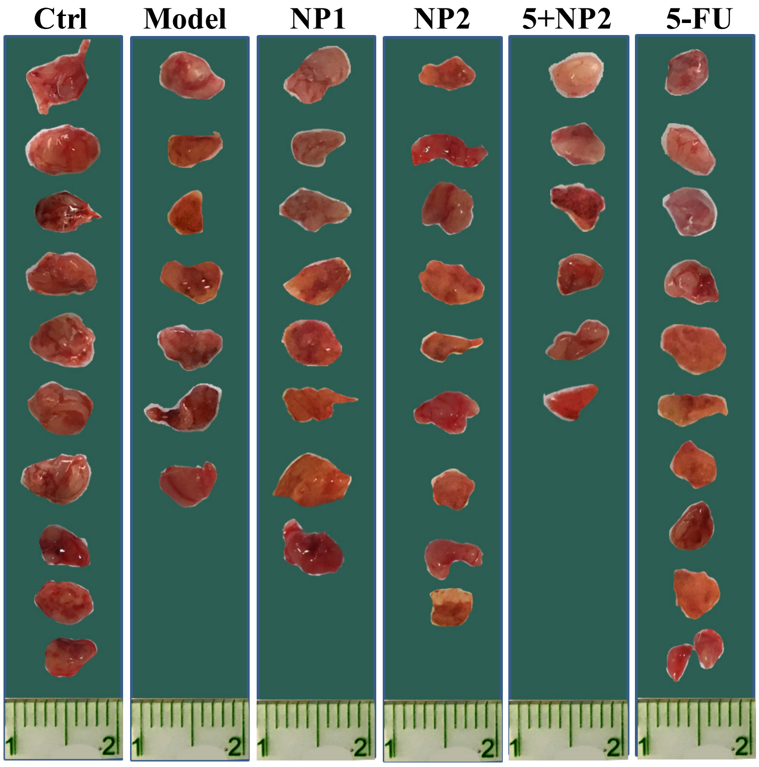

Figure S5 : Morphology of the tumor (A) and spleen tissues (B) kidney (C) and thymus (D) of each group taken out from the sacrificed mice at the study end point.

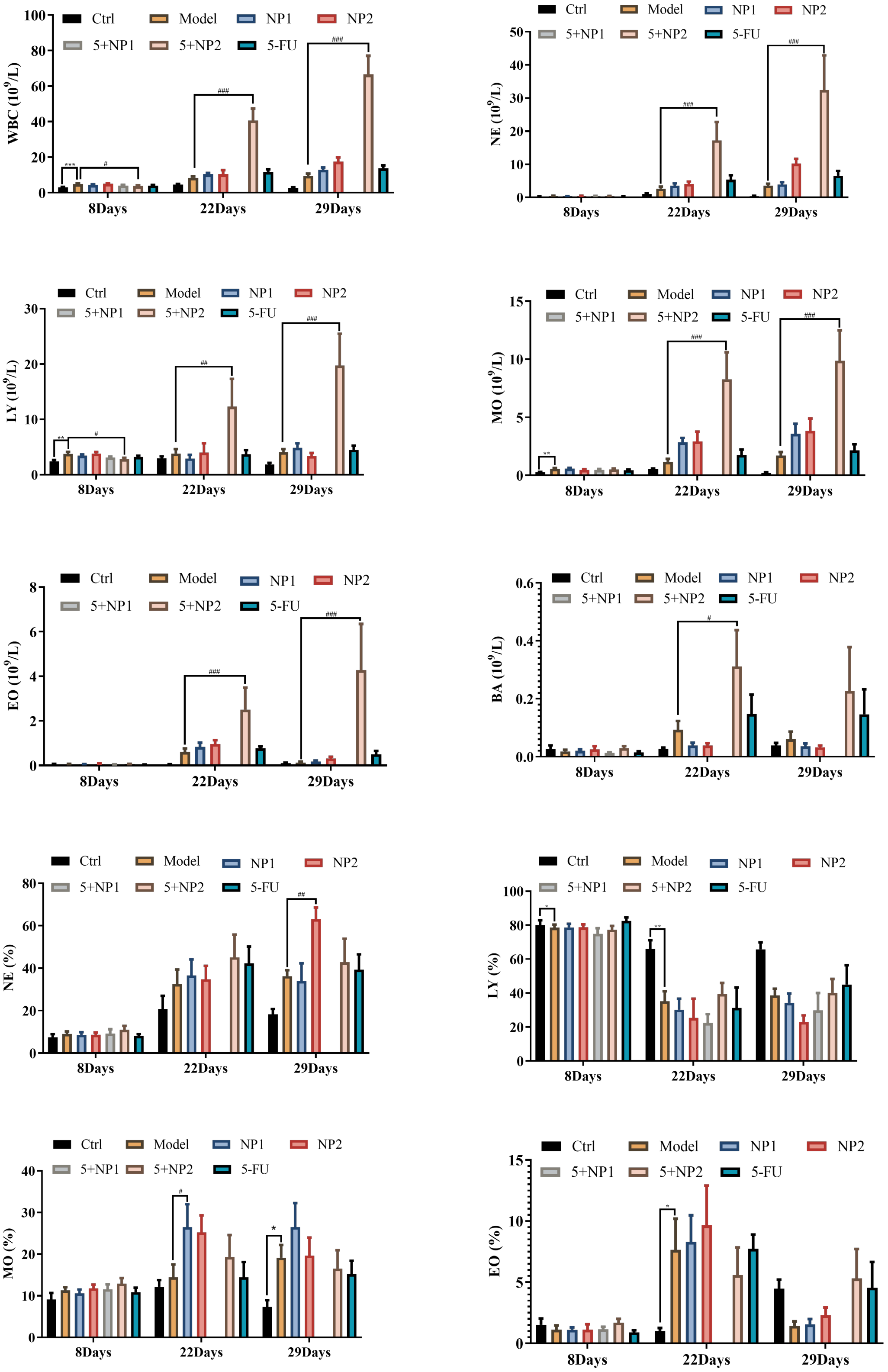
Ctrl
Model
NP1
NP2
5+NP1
5+NP2
5-FU
WBC (10^9/L)
NE (10^9/L)
LY (10^9/L)
MO (10^9/L)
EO (10^9/L)
BA (10^9/L)
NE (%)
LY (%)
MO (%)
EO (%)
8Days
22Days
29Days

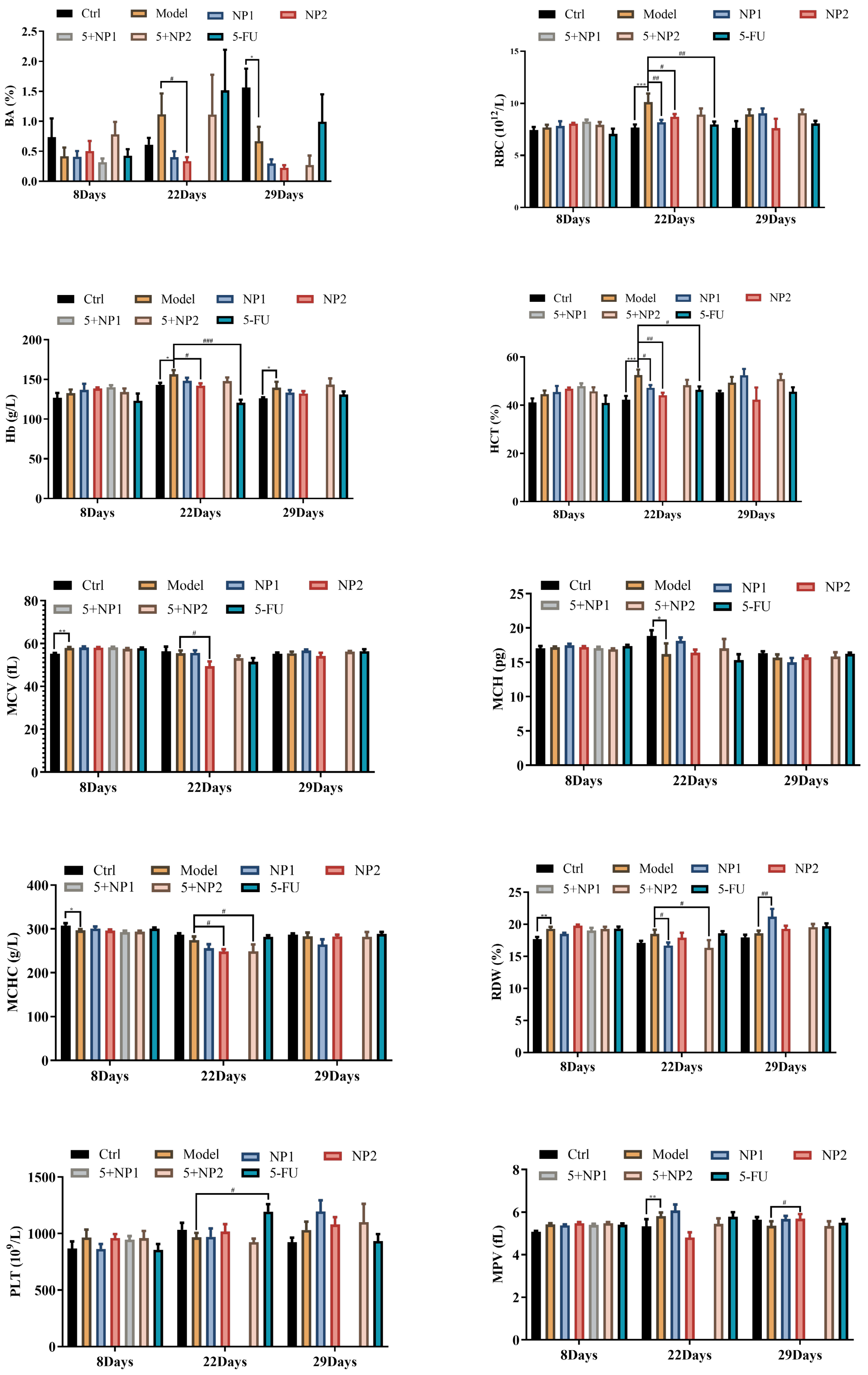

Figure S6. Effect of NP1 and NP2 on blood cell in liver mice with HEP1-6 cells injection Values wereshown as Mean±SEM. **p*<0.05 ,***p*<0.01 and ****p*<0.001 vs sham group ; #*p*<0.05, ##*p*<0.01 and###*p*<0.001 vs model group.
